# The Luteovirus P4 Movement Protein Is a Suppressor of Systemic RNA Silencing

**DOI:** 10.3390/v9100294

**Published:** 2017-10-10

**Authors:** Adriana F. Fusaro, Deborah A. Barton, Kenlee Nakasugi, Craig Jackson, Melanie L. Kalischuk, Lawrence M. Kawchuk, Maite F. S. Vaslin, Regis L. Correa, Peter M. Waterhouse

**Affiliations:** 1 School of Life and Environmental Sciences, University of Sydney, Sydney, NSW 2006, Australia; adriana.fusaro1@gmail.com (A.F.F.); deborah.barton@sydney.edu.au (D.A.B.); knakasugi@illumina.com (K.N.); craig.jackson@sydney.edu.au (C.J.); mkalischuk@ufl.edu (M.L.K.); lawrence.kawchuk@agr.gc.ca (L.M.K.); 2 Plant Industry Division, CSIRO, P.O. Box 1600, Canberra, ACT 2601, Australia; 3 Department of Virology (M.F.S.V.), Department of Genetics (R.L.C.) and Institute of Medical Biochemistry (A.F.F.), Federal University of Rio de Janeiro (UFRJ), Rio de Janeiro 21941-590, Brazil; maite@micro.ufrj.br; 4 North Florida Research and Education Center, University of Florida, Quincy, FL 32351, USA; 5 Department of Agriculture and Agri-Food Canada, Lethbridge, AB T1J4B1, Canada; 6 School of Earth, Environmental and Biological sciences, Queensland University of Technology, Brisbane, QLD 4001, Australia

**Keywords:** *Luteoviridae*, *Luteovirus*, *Polerovirus*, RNA silencing suppression

## Abstract

The plant viral family *Luteoviridae* is divided into three genera: *Luteovirus*, *Polerovirus* and *Enamovirus*. Without assistance from another virus, members of the family are confined to the cells of the host plant’s vascular system. The first open reading frame (ORF) of poleroviruses and enamoviruses encodes P0 proteins which act as silencing suppressor proteins (VSRs) against the plant’s viral defense-mediating RNA silencing machinery. Luteoviruses, such as barley yellow dwarf virus-PAV (BYDV-PAV), however, have no P0 to carry out the VSR role, so we investigated whether other proteins or RNAs encoded by BYDV-PAV confer protection against the plant’s silencing machinery. Deep-sequencing of small RNAs from plants infected with BYDV-PAV revealed that the virus is subjected to RNA silencing in the phloem tissues and there was no evidence of protection afforded by a possible decoy effect of the highly abundant subgenomic RNA3. However, analysis of VSR activity among the BYDV-PAV ORFs revealed systemic silencing suppression by the P4 movement protein, and a similar, but weaker, activity by P6. The closely related BYDV-PAS P4, but not the polerovirus potato leafroll virus P4, also displayed systemic VSR activity. Both luteovirus and the polerovirus P4 proteins also showed transient, weak local silencing suppression. This suggests that systemic silencing suppression is the principal mechanism by which the luteoviruses BYDV-PAV and BYDV-PAS minimize the effects of the plant’s anti-viral defense.

## 1. Introduction

Plants fight against viral infection with their intrinsic RNA degradation mechanism, often termed the RNA silencing machinery [1]. In *Arabidopsis thaliana*, virus-derived double-stranded RNA (dsRNA) molecules are targeted by RNase III-family DICER-LIKE (DCL) proteins, generating 21 to 24 nucleotide (nt)-long small interfering RNAs (siRNAs) [2,3,4]. Most RNA viruses are preferentially degraded by the DCL4 protein, which explains the greater accumulation of 21-nt siRNAs in most of the plants analyzed so far [5]. DCL2- and DCL3-produced siRNAs of 22 and 24 nt, respectively, may also play important antiviral roles [2,3,4,6,7]. Virus-derived siRNAs of all size classes guide distinct ARGONAUTE (AGO) proteins to single-stranded RNA molecules, promoting their destabilization [8]. The efficiency of this antiviral defense system is also largely dependent on a siRNA amplification step promoted by host RNA-dependent RNA polymerases (RDRs). Following AGO-mediated cleavage, RDRs, in particular RDR1 and RDR6, use target viral RNAs to make new dsRNA molecules which are, in turn, also processed by DCLs, generating secondary virus-derived siRNAs [6,9]. Secondary siRNAs may presumably act as systemic silencing signals, priming antiviral responses in uninfected cells [10]. Some viruses, on the other hand, are able to counteract this defense system by expressing proteins that act as viral suppressors of RNA silencing (VSRs). Two major strategies used by VSRs are known: inhibiting the production or accumulation of locally-acting siRNAs in the infected tissue (local suppression) or preventing the spread of silencing signals to systemic leaves (systemic silencing suppression) [11]. Some VSRs bind to the siRNAs directing the silencing machinery (e.g., tombusvirus P19) [12], while others target one or several proteins of the RNA silencing pathway, like ARGONAUTE1 (AGO1) (targeted by cucumovirus 2b) [13], DCL4 (targeted by carmovirus P38) [3] and DRB4 (targeted by caulimovirus P6) [14].

**Figure 1:**
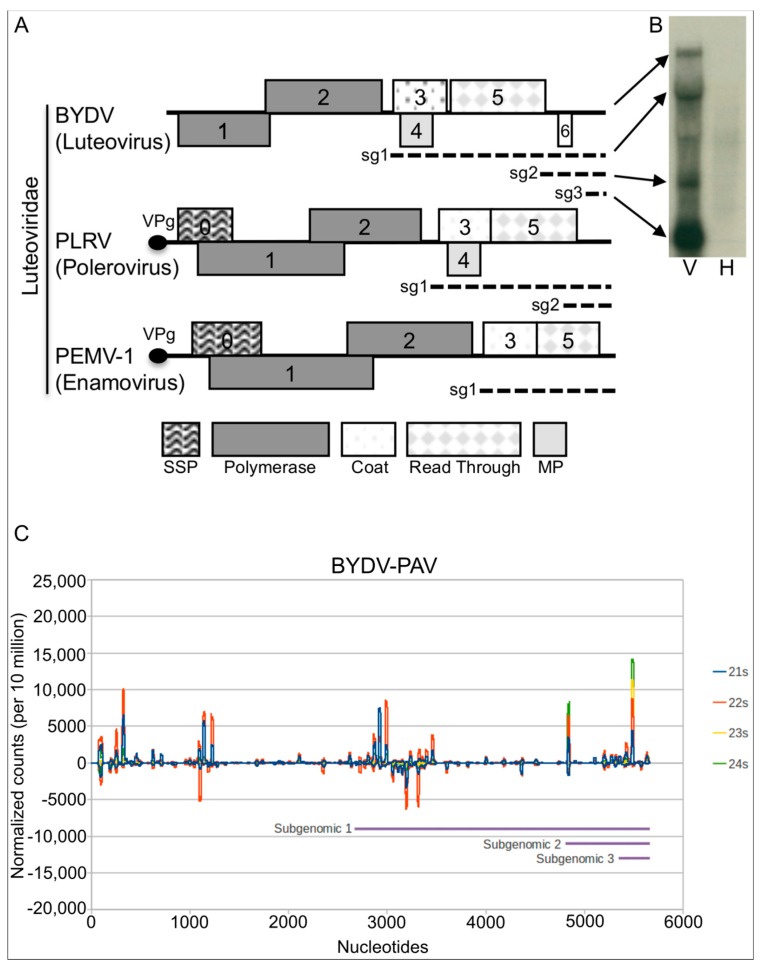
Genome organization of the family *Luteoviridae* and mapping of perfect match 21 to 24 nt virus-derived small RNAs from barley yellow dwarf virus-PAV (BYDV-PAV)-infected plants. (**A**) Genome organization of viruses belonging to the three genera of the family *Luteoviridae*. Numbers within rectangles indicate known luteovirid ORFs: silencing suppressor protein (VSR), polymerase, coat-protein, read-through transmission protein and movement protein (MP). Subgenomic RNAs (sg1, sg2 and sg3) are represented as dotted lines. (**B**) Northern blot from virus-infected (V) and healthy (H) plants showing the accumulation of genomic and subgenomic BYDV-PAV RNAs (reproduction from Kelly et al. 1994 [33]). (**C**) Profiles of 21 to 24 nt size reads mapping to the BYDV-PAV genome deep-sequenced from barley-infected plants. Profile is shown as a cumulative number of reads per nucleotide, normalized to the total library size per 10 million reads. Reads that aligned to the negative strand are plotted as negative Y-axis values. Purple bars show the sub-genomic RNA regions (positions starting at 2678, 4817 and 5356). 'Hot-spots' or peaks of read counts can be seen across the genome.

The members of the family *Luteoviridae* (luteovirids) are divided into three genera: *Luteovirus*, *Polerovirus* and *Enamovirus*. They are transmitted by aphids in a persistent manner, and are restricted to the phloem tissue where a number of them cause cell necrosis. Their single stranded RNA genomes each contain 5–6 major open reading frames (ORFs) and produce subgenomic RNAs as part of their gene expression strategy (Figure 1A). There is clear sequence conservation in ORFs 3, 4 and 5 among all of the family members. The major capsid protein is encoded by ORF 3 [15,16], and ORF 4, which is absent in the enamovirus pea enation mosaic virus-1 (PeMV-1), encodes a cell-to-cell movement protein [17]. A small ORF upstream of ORF 3, named ORF 3a, was recently discovered in luteovirids and its corresponding P3a protein is involved in systemic virus movement in the polerovirus turnip yellows virus, with a possible role in cap-independent translation in luteoviruses [18]. The protein generated by translational readthrough from ORF 3 into ORF 5 is required for efficient aphid transmission [19]. There is considerable sequence diversity in the 5' portion of the genomes (ORFs 0, 1 and 2) of the luteovirids, but the P0 proteins, encoded by ORF 0 of the poleroviruses and enamovirus, have the conserved function of being VSRs that target AGO1 for degradation [11,20].

Luteoviruses, such as barley yellow dwarf virus-PAV (BYDV-PAV), have no P0 to carry out the VSR role, so we investigated whether other proteins or RNAs encoded by BYDV-PAV confer protection against the plant’s silencing machinery. We were unable to detect any evidence of protection afforded by a possible decoy effect of the highly abundant subgenomic RNA3, nor from proteins encoded by ORFs 1, 2, 3 or 5. However, we detected a strong suppression of systemic silencing by P4 and a similar, but weaker, activity by P6. The BYDV-PAS P4, but not the polerovirus potato leafroll virus (PLRV) P4, also displayed a similar VSR activity. Both luteovirus and the polerovirus P4 proteins also showed transient, weak local silencing suppression. Transient expression of luteovirus P4 in *Nicotiana benthamiana* triggered dose-dependent cell necrosis which increased over time, but it did not inhibit P4’s systemic VSR activity against the propagation and spread of anti-viral siRNAs, nor did it prevent the replication and egress of potato virus X (PVX). This suggests that systemic silencing suppression is the principal mechanism by which the luteoviruses BYDV-PAV and BYDV-PAS minimize the effects of the plant’s anti-viral defense.

## 2. Materials and Methods

### 2.1. Sequence Analysis

The accession numbers of amino acid sequences used in multiple alignments were obtained from NCBI and are as follows: barley yellow dwarf virus—PAV (NC_004750) containing protein coding sequences NP_840067, NP_840014, NP_840015, NP_840016, NP_840017, NP_840018; pea enation mosaic virus (NC_003629) containing protein coding sequences NP_619735, NP_620026, NP_619736, NP_620027, NP_619738; beet western yellows virus P0, NP_840095; cucurbit aphid-borne yellows virus P0, NP_620100; cereal yellow dwarf virus-RPV P0, NP_840020; potato leafroll virus P0, NP_056746; sugarcane yellow leaf virus P0, NP_050005; cocksfoot mottle virus P1, NP_941375; rice yellow mottle virus P1, NP_041737; southern bean mosaic virus P1, NP_660270; cymbidium ringspot virus P19, CAA33535; turnip crinkle virus P38, NP_620723; tobacco etch virus HCPro, AAA47910; cucumber mosaic virus 2b, AEB39608; BYDV-PAS P4, AAF26426.1; PLRV P4, D13953.1; BYDV-GAV P4, AAO65189.1; BYDV-PAV P6, APD77443.1; BYDV-GAV P6, AAO65190.1; BYDV-PAV isolate 064 P6, ABP68814.1; BYDV-PAV isolate 047 P6, ABP68742.1; BYDV-PAV isolate 048 P6, ABP68754.1; BYDV-PAV isolate 052 P6, ABP68760.1; BYDV-PAV isolate 05GG6 P6, ABY73569.1; BYDV-PAV isolate 04ZZ5 P6, ABY73551.1. Multiple sequence alignments using default parameters and percent identities were calculated using ClustalX 2.0.12 [21]. Phylogenetic analyses were conducted using MEGA version 5.0 [22]. Consensus phylogenetic trees were constructed by the neighbour-joining method with pairwise deletion and bootstrap with 1000 replications.

### 2.2. Small RNA Deep Sequencing and Bioinformatic Analyses

Total RNA was extracted from barley plants infected with BYDV-PAV using the miRNeasy kit (Qiagen, Hilden, Germany), according to the manufacturer’s instructions. BYDV-PAV small RNA library was prepared (Illumina small RNA sample preparation kit, Illumina, San Diego, CA, USA) and deep sequenced on the Illumina GAIIx platform (Illumina, San Diego, CA, USA) according to the manufacturer's protocols. Deep sequencing reads were preprocessed (read quality assessment, adapter removal, read size extraction) utilizing the Fastx Toolkit set of scripts (http://hannonlab.cshl.edu/fastx_toolkit/index.html) and in-house custom perl scripts. Small RNA reads between 21 and 24 nt were mapped to the BYDV-PAV reference genome using Bowtie v0.12.7 [23], with no mismatches allowed. Read distribution profile visualization and coverage analyses were performed utilizing custom perl scripts, mpileup from SAMtools [24], and the OpenOffice (in Ubuntu Linux) spreadsheet application. For comparisons of read distribution profiles, reads were normalized against the total read count (after preprocessing) per 10 million reads, and plotted as number of reads per base.

### 2.3. DNA Constructs

Constructs pBin-35S-mGFP5 [25], pJL3:P19 [26], *L6MHV* encoding an autoactive allele of the flax *L6* rust resistance gene [27], and PVX-GFP, P0^PL^, P0^CY^ and P1^RY^ [20] were described previously. Binary vector pPTN253 encoding *CED-9* from *Caenorhabditis elegans* [28] driven by the 35S cauliflower mosaic virus (CaMV) promoter was a gift by James Dale (QUT, Australia).

The cDNA of ORFs of BYDV-PAV P1-P2 (NP_840014), P2 (NP_840067), P3 (NP_840015), P4 (NP_840016), P3-P5 (NP_840017) were synthesized by GENEART (Regensburg, Germany) and transferred from the pUC vector to the CaMV 35S-expression cassette in the pART7 vector [29]. Each 35S-expression cassette was then transferred as a NotI fragment into the binary vector pBART. For the analysis of different P4 proteins and P6, the cDNA of ORFs of BYDV-PAV P4 (NP_840016), PLRV P4 (D13953.1) and BYDV-PAV P6 (APD77443.1) were PCR-amplified with primers that introduced a HindIII site at the 5′ end and an XbaI site at the 3′ end of each ORF. P4^PAV^ cDNA was amplified with primers 5′-GAATTCAAGCTTACCATGGCACAAGAAGGAGG-3′ (forward) and 5′-GAATTCTCTAGACTATCGTTGATTCCTGGA-3′ (reverse); P4^PL^ cDNA was amplified with primers 5′-GAATTCAAGCTTACCATGTCAATGGTGGTGCAC-3′ (forward) and 5′-GAATTCTCTAGATCATCCGCGCTTGATAG-3′ (reverse), and P6^PAV^ cDNA was amplified with primers 5′-GAATTCAAGCTTATGGATGACCTCCACGTTATC-3′ (forward) and 5′-GAATTCTCTAGATTAAACAGAAGAGCGGAAGGAG-3′ (reverse). Following digestion of PCR products, each ORF was cloned as a HindIII-XbaI fragment directly into the pORE1 binary expression vector [30] containing a CaMV 35S expression cassette, generating constructs pORE1-35S::PAV4, pORE1-35S::PAV6 and pORE1-35S::PLRV4. The cDNA of BYDV-PAS P4 (AAF26426.1) was synthesized and cloned into a pUC plasmid by GENEART (Regensburg, Germany) with unique HindIII and XbaI sites as 5′ and 3′ flanking sequences, respectively. Following plasmid digestion, the HindIII-XbaI fragment corresponding to the P4^PAS^ ORF was ligated into the pORE1 binary vector (mentioned above) to generate pORE1-35S::PAS4.

### 2.4. Transient Expression Assay in N. benthamiana

Transgenic *N. benthamiana* (line 16c), which is homozygous for the GFP transgene [25], and the *Agrobacterium* infiltration method have been described previously [31]. For co-infiltrations of wild-type *N. benthamiana* or line 16c, cultures of *A. tumefaciens* (strain GV3101) harboring a relevant binary plasmid, were mixed before infiltration to a final OD_600_ = 0.5 each, except for L6 and CED-9 (OD_600_ = 1). Dilutions of P4^PAV^, P4^PAS^ and P4^PL^ were obtained by adding appropriate concentrations of a vector-less *Agrobacterium* culture to the infiltrate solutions. The GFP fluorescence was monitored with a hand-held UV lamp as previously described [31]. Pictures (UV and visible light) were taken with a Canon EOS 550D digital camera (Canon, Tokyo, Japan).

### 2.5. RNA Blot Analysis

Total RNA was extracted from agroinfiltrated leaves of *N. benthamiana* 16c collected at 6 dpi, with TRIzol reagent (Invitrogen, Carlsbad, CA, USA). The manufacturer’s protocol was modified as an additional phenol:chloroform wash was added before an overnight precipitation step at −20 °C. For high and low molecular weight Northern blots, 20 µg and 40 µg of total RNAs were loaded and run on formaldehyde and polyacrylamide gels, respectively. RNA detection and hybridization conditions were previously described [20].

## 3. Results

### 3.1. BYDV-PAV Elicits an RNA Silencing Response in the Phloem

BYDV-PAV is known to circularize its RNA for cap-independent translation of its genome [32]. It also produces a highly abundant non-coding sgRNA3 (Figure 1B) [33]. We wondered if, in the absence of a P0 VSR, these attributes might in some way protect BYDV-PAV against the plant’s anti-viral RNA degradation system in the phloem. Therefore, we examined the small RNA (sRNA) profiles of virus-infected plants. Total RNA from BYDV-PAV-infected barley was extracted, size-fractionated, and the sRNAs sequenced using an Illumina machine. The predominant class of sRNAs, identical or complementary in sequence to the BYDV-PAV genome, was 22 nt (502k reads) with less abundant 21 nt (328k reads) and 24 nt (146k reads) size classes (Figure 1C). This profile of abundant siRNAs (representing 9.76% of total sRNAs) shows that the silencing machinery in phloem tissues mounts a response to BYDV-PAV infection, suggesting that circularization of BYDV-PAV genomic RNA for translation does not strongly inhibit this process, as shown for other viruses (e.g., [34]). Despite the abundance of sgRNA3, which might have acted as a decoy to saturate the plant’s silencing machinery and thereby protect the viral genome from degradation, there did not appear to be a predominance of siRNAs from this region, suggesting that BYDV-PAV employs a different silencing suppression or avoidance strategy.

### 3.2. Assaying BYDV-PAV-Encoded Proteins for Silencing Suppressor Activity

In an attempt to identify potential VSR candidates in BYDV-PAV, we compared its ORF sequences with those of known suppressors, including rice yellow mottle virus (RYMV) P1, tomato bushy stunt virus (TBSV) P19, turnip crinkle virus (TCV) P38, cucumber mosaic virus (CMV) 2b, potato virus Y (PVY) HCPro and five P0s (data not shown). We also compared the translated ORF sequences using TBLASTn and BLASTp against accessions in the NCBI plant viral database containing the keyword “suppressor”. Neither analysis produced any obvious VSR ortholog for any of the six BYDV ORFs. Therefore, the proteins P1-2, P2, P3, P4, P3-5 and P6 encoded by the BYDV-PAV genome were tested for local and systemic VSR activity using the 16c system [35]. The virus genes were placed under the control of the 35S promoter in binary vectors and transiently co-expressed with a sense GFP transgene (sGFP) in fully-expanded leaves of *N. benthamiana* line 16c. This line has an integrated GFP transgene [25,31] and is a well stablished assay for detecting suppression activity, as a bright green fluorescent tissue is observed under the UV light 3–7 days post infiltration (dpi) if the candidate protein is able to block the locally-induced silencing triggered by the sGFP transgene. If the co-infiltrated virus gene has no systemic VSR activity, the infiltrated tissue emits a signal that spreads through the phloem and emerges in the apex and young leaves of the plant to silence the GFP; co-infiltration with a systemic VSR delays or negates the emergence of distal silencing. The percentage of plants that fail to show distal silencing after 2–3 weeks is taken as a measure of the candidate genes’ systemic VSR activity [36]. 

**Figure 2:**
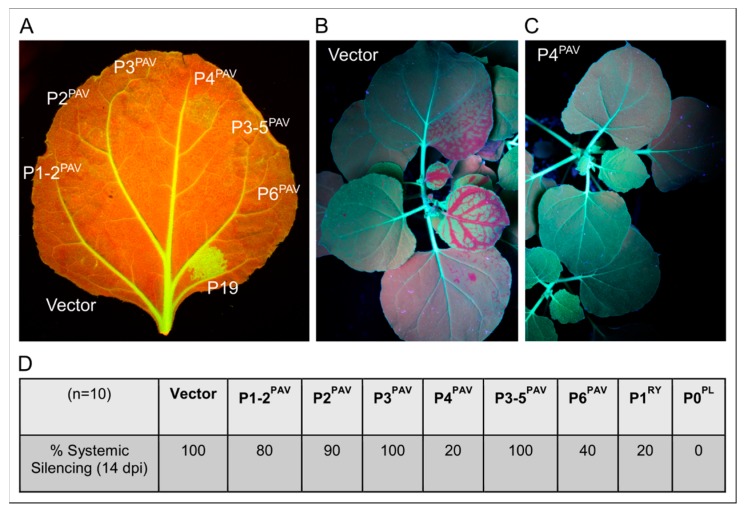
Local and systemic silencing suppression assays of luteovirus BYDV-PAV proteins. (**A**) Agro-infiltration of GFP-expressing *N. benthamiana* 16c leaves with sense GFP transgene plus BYDV-PAV proteins P1-2, P2, P3, P4, P3-5 or P6. The empty vector was used as a negative control for silencing suppression activity, while P19 from tomato bushy stunt virus (TBSV) was used as a positive control. Pictures were taken at 5 dpi under UV light. (**B**) Systemic silencing assay control. *N. benthamiana* 16c plants were agro-infiltrated with a sense GFP inducer plus the empty vector pBART. Picture of whole plants showing systemic silencing was taken at 14 dpi under UV light. (**C**) Systemic silencing assay for the BYDV-PAV P4 protein. *N. benthamiana* 16c plants were agro-infiltrated with a sense GFP inducer plus the P4 protein. Picture of whole plants showing no systemic silencing was taken at 14 dpi under UV light. (**D**) Scores of silencing suppression activity for the BYDV-PAV proteins P1-2, P2, P3, P4, P3-5 and P6. The P1 protein from RYMV and the P0 protein from PLRV were used as positive controls.

Using this system and the well-described local VSR, P19, from TBSV as a control, BYDV-PAV P1-2, P2, P3, P3-5 and P6 showed little or no evidence of local VSR activity at 6 dpi (Figure 2A), even when multiple proteins were co-expressed (data not shown). This contrasts with a report that P6 from BYDV-GAV has local VSR activity [37], but this may be due to the marked sequence divergence of GAV from other BYDV isolates, especially in the P6 ORF sequence (Figure S1). BYDV-PAV P4 (P4^PAV^), however, showed a weak, transient local VSR activity that could be detected through a mild increase in GFP fluorescence when compared to control empty vector. Assessing the spread of the silencing signal from infiltrated spots at 14 dpi revealed that P4^PAV^ and BYDV-PAV P6 (P6^PAV^) produce strong and moderate systemic silencing suppression, respectively, when compared to the well-known systemic VSR P1 from RYMV (P1^RY^) (Figure 2B–D). The spread of the silencing signal was detected in only 20% of plants co-infiltrated with P4^PAV^, a profile of systemic VSR activity equivalent to that of P1^RY^, while 40% of plants showed systemic silencing when co-infiltrated with P6^PAV^. This same moderate systemic VSR activity has also been reported for BYDV-GAV P6 [37].

**Figure 3:**
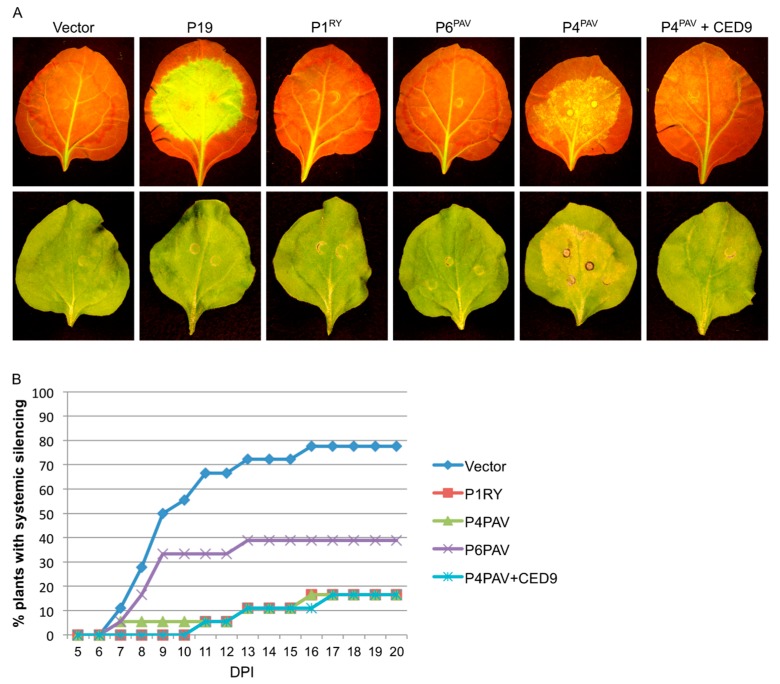
Inhibition of necrosis does not affect BYDV-PAV P4 local and systemic silencing suppression activities. (**A**) *N. benthamiana* 16c plants were agro-infiltrated with a sense GFP inducer plus the empty vector pBART, TBSV P19, RYMV P1 (P1^RY^) or the BYDV-PAV proteins P6^PAV^ or P4^PAV^. The P4^PAV^ was also agro-infiltrated in the presence of the antiapoptotic protein CED-9 from *C. elegans* for inhibition of P4^PAV^-induced necrosis on the infiltrated tissue. The effects of the different suppressors on local cell-to-cell silencing spread can be analysed by the dynamics of the red halo formation around the infiltrated area. Pictures were taken at 7 dpi under UV (upper panel) or normal light (lower panel). (**B**) Plants (*n* = 18) infiltrated with the above constructs were scored for the appearance of systemic silencing at different days post-infiltration (DPI).

When analyzing the short-distance spread of the silencing signal at 7 dpi, it became apparent that P4^PAV^, but not P6^PAV^, reduces the development of the red “halo” (Figure 3A) which normally reports the initial cell-to-cell spread of the mobile silencing signal to adjacent cells outside the infiltrated area [38,39,40]. This result shows that the VSR activity by P4^PAV^ delays the cell-to-cell movement of the systemic silencing signal. This assay also showed that P4^PAV^, but not P6^PAV^, induces necrosis of the infiltrated tissue, which, under the blue light used to excite GFP, gives a yellowish fluorescence making it difficult to visually assess the GFP expression. The necrosis induced by P4^PAV^ could be largely prevented by adding a CED-9-encoding construct to the P4^PAV^ treatment (Figure 3A), since CED-9 is an antiapoptotic protein from *C. elegans* shown to suppress viral-induced necrosis in plants [41]. Inhibition of P4^PAV^-triggered necrosis by CED-9 did not impact on P4^PAV^ VSR activity, since the delay of red halo formation at 7 dpi or 11 dpi (Figure 3A and Figure S2), or the strong suppression of systemic silencing by P4^PAV^ (Figure 3B), remained unaffected in plants agro-infiltrated with a mix containing P4^PAV^ and CED-9 constructs. These results suggest that the VSR activity by P4^PAV^ and induction of necrosis are not linked.

### 3.3. Systemic Silencing Suppression and Necrosis Is Produced by P4 from Two Different Luteoviruses, but Not by P4 from a Polerovirus

**Figure 4:**
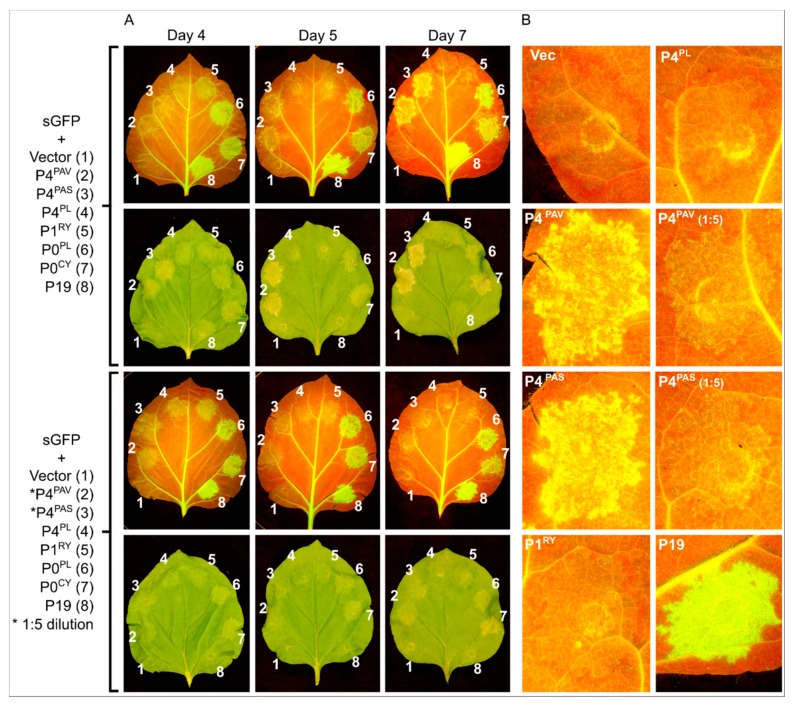
Induction of necrosis by different luteovirus P4 proteins is dose-dependent. Agro-infiltration of GFP-expressing *N. benthamiana* 16c leaves with sense GFP transgene (sGFP) plus the BYDV-PAV P4 (2), BYDV-PAS P4 (3), PLRV P4 (4) or the control empty pBART vector (1), RYMV P1 (5), PLRV P0 (6), CYDV P0 (7) or TBSV P19 (8) ((**A**), top panel). Necrosis symptoms were significantly lessened when the P4 from BYDV-PAV and BYDV-PAS was tested at a five-fold dilution (0.1 OD) ((**A**), bottom panel, see asterisk). Pictures were taken at 4, 5 and 7 dpi under UV or white light. (**B**) Close-up of selected infiltrations at 7 dpi highlighting that the P4 from BYDV-PAV and BYDV-PAS, but not the P4 from PLRV are able to inhibit the formation of a red halo around the infiltrated area, a hallmark of the cell-to-cell silencing spread.

To explore whether P4 proteins in other luteoviruses and poleroviruses also have VSR activity, the appropriate ORF was cloned from BYDV-PAS (P4^PAS^) and from PLRV (P4^PL^), and placed under the control of the CaMV 35S promoter. Testing the constructs in the 16c system with the standard agro-inoculum strength of 0.5 OD (Figure 4A top and Figure 4B) showed that P4^PAS^, but not P4^PL^, induced a delay in red halo formation and onset of necrosis in a very similar way to P4^PAV^. With the 0.5 OD agro-inoculum, the necrosis induced by P4^PAV^ or P4^PAS^ was barely discernable to the naked eye at 4 dpi and obvious at 7 dpi (Figure 4A top and Figure 4B), and the boundaries of the spots were as devoid of red halos as the P19 spots. Diluting the P4^PAV^ and P4^PAS^ infiltrate solutions five-fold (Figure 4A bottom and Figure 4B 1:5 dilution), with buffer containing vector-less agrobacterium, led to some halo induction and reduction of necrosis, which indicates a dose-dependent effect by both P4 proteins on red halo formation and necrosis induction. The five-fold dilution of P4^PAV^ and P4^PAS^ infiltrate solutions also produced a mild increase in GFP fluorescence in infiltrated spots at 4 dpi when compared to the control empty vector, and this difference to the control was still discernable at 7 dpi, although less obvious. This mild increase in GFP fluorescence compared to the control was also noted in spots infiltrated with P4^PL^ at 4 dpi, but no longer detectable at 5 dpi (Figure 4A top and bottom). These results indicate that both P4^PAV^ and P4^PAS^ produce weak and transient local VSR activity, which is even less pronounced in the case of P4^PL^.

**Figure 5:**
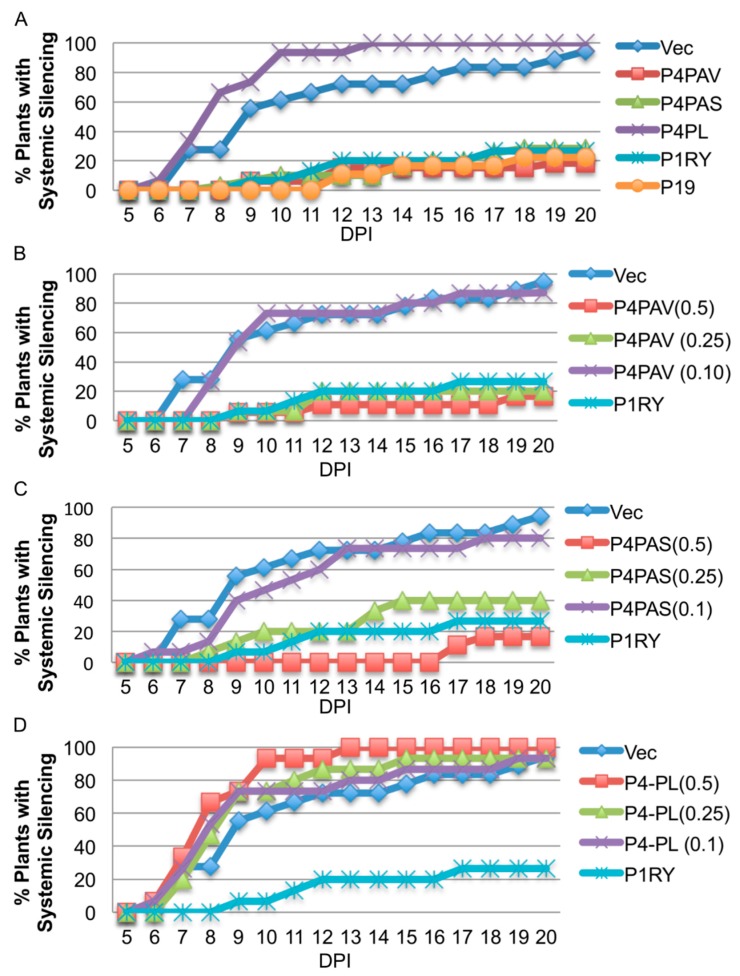
Dose-dependent systemic silencing suppression activities of the luteovirus P4 proteins. *N. benthamiana* 16c plants were agro-infiltrated with a sense GFP transgene plus the empty vector (vec), BYDV-PAV P4 (P4PAV), BYDV-PAS P4 (P4PAS), PLRV P4 (P4PL), RYMV P1 (P1RY) or TBSV P19. Percentage of plants showing systemic silencing was scored at different days post-infiltration (DPI). (**A**) Agro-infiltration of 0.5 OD cultures of all candidate suppressor proteins. (**B**) Agro-infiltration of 0.5, 0.25 or 0.1 OD cultures of P4PAV. (**C**) Agro-infiltration of 0.5, 0.25 or 0.1 OD cultures of P4PAS (**D**) Agro-infiltration of 0.5, 0.25 or 0.1 OD cultures of P4PL. The empty vector, P19 and P1RY at 0.5 OD were used as negative and positive controls.

Single leaves of young 16c plants were infiltrated with a cocktail of sGFP and one of the P4 or control vectors, and observed for 20 days for the emergence of systemic silencing. P4^PAV^ and P4^PAS^, but not P4^PL^, gave profiles of strong systemic suppressors (Figure 5A), and this lack of VSR activity by P4^PL^ (Figure 5A,D) could be explained by the low sequence conservation (~53%) between the polerovirus P4 and the two luteovirus P4 proteins which are highly conserved (~91% sequence identity) (Figure S1). In this experiment, both P4^PAV^ and P4^PAS^ showed stronger VSR activities than P1^RY^, and using different agro-infiltrate concentrations of the P4 constructs revealed that P4^PAS^ is slightly weaker than P4^PAV^ (Figure 5B,C), with their systemic VSR activities being dose-dependent.

### 3.4. P4^PAV^ and P4^PAS^ Reduce the Production or Accumulation of siRNAs

**Figure 6:**
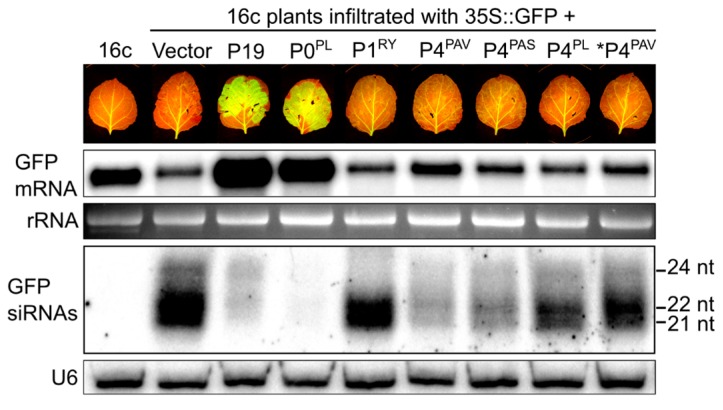
P4^PAV^ and P4^PAS^ reduce the accumulation of siRNAs. GFP-expressing *N. benthamiana* 16c leaves were co-infiltrated with sense GFP plus the empty vector, TBSV P19, PLRV P0 (P0^PL^) or RYMV P1 (P1^RY^) as controls, or BYDV-PAV P4 (P4^PAV^), BYDV-PAS P4 (P4^PAS^) or PLRV P4 (P4^PL^). P4^PAV^ was also infiltrated at a five-fold dilution (*P4^PAV^). Non-infiltrated plants (16c) were also included as negative controls. Pictures were taken under the UV light and leaf samples collected for Northern blots at 6 dpi. Total RNA (20 μg) was blotted and probed for the presence of GFP mRNA. rRNA was used as a loading control for high-molecular-weight RNA. Total RNA (40 μg) from the agro-infiltrated tissues was also blotted and probed for the presence of GFP siRNAs. U6 was used as a loading control for low-molecular weight RNA. RNA size markers are indicated.

To investigate the mechanism of the systemic VSR activity of P4^PAV^ and P4^PAS^, the production of siRNAs was measured by Northern blots. Agroinfiltration of 16c plants with the 35S:GFP transgene is known to induce the production of siRNAs against the GFP mRNA by an RNA-dependent RNA polymerase-mediated process and these secondary siRNAs guide the destruction of the GFP mRNA [42,43]. Total RNA extracts were made from 16c leaves infiltrated with different cocktails of agrobacterium cultures containing constructs for the expression of GFP and candidate or control VSR genes, and collected at 6 dpi. The RNA extracts were size fractionated, run on gels, transferred to membranes and probed with the 300-nt 3′ sequence of the GFP gene. The blots (Figure 6) show that the GFP mRNA level dropped from its endogenous level (16c) in tissues infiltrated with the 35S:GFP transgene (Vec) and became elevated in leaf tissues co-infiltrated with the transgene and P19 or P0^PL^. Tissues co-infiltrated with 35S:GFP and P1^RY^, P4^PAS^, or P4^PL^ showed GFP mRNA levels similar to those of the GFP transgene alone. Interestingly, P4^PAV^-infiltrated tissues had slightly increased GFP mRNA accumulation, which could be almost nullified by a five-fold dilution of the P4^PAV^ agro-inoculum concentration, confirming the weak local VSR activity by P4^PAV^. As expected, the enhancement of GFP mRNA levels by P19 and P0 were mirrored by reduced levels of GFP-specific siRNAs. This is consistent with their local VSR activities, since P19 affects siRNA stability and production, while P0 affects the production of siRNAs [12,20,44,45]. The siRNA levels of all size classes were also considerably reduced in the tissues infiltrated with P4^PAV^ and P4^PAS^, with a minor reduction in tissues treated with P4^PL^ or the diluted P4^PAV^ inoculum.

The visible GFP fluorescence of P4^PAV^ and P4^PAS^ infiltrated leaves (Figure 6) and the GFP mRNA levels in samples taken from them (Figure 6, GFP mRNA blot) were not greatly elevated, unlike those treated with P19 or P0. However, the GFP-specific siRNAs in the P4^PAV^ and P4^PAS^ samples were nearly as low as in the P19 or P0 treated samples (Figure 6, siRNA blot). This suggests that systemic VSR activities of P4^PAV^ and P4^PAS^ are related to their capacity to reduce the local production or accumulation of secondary siRNAs, which could be explained, in part, by their weak local VSR activities. However, P4^PAV^ and P4^PAS^ probably operate differently to either P19 or P0 since they lack strong local silencing suppression activity. The different increase in GFP mRNA accumulation at 6 dpi (Figure 6, GFP mRNA blot) confirms that P4^PAS^ is slightly weaker than P4^PAV^ in its VSR activity.

### 3.5. Induction of Cell Death by P4^PAV^ and P4^PAS^ Does Not Prevent Viral Replication and Spread

As both BYDV and the only other described luteovirus, bean leafroll virus, are known to induce phloem necrosis in their hosts [46,47,48], it is possible that the P4s of these viruses could be the elicitors of this response. This suggests that these viruses would have evolved to not only fight the anti-viral silencing machinery but to also escape from cells undergoing host-induced cell death in the vasculature. For this to be true, the virus has to replicate and egress from the infected cell before the cell dies from necrotic processes induced by P4. 

**Figure 7:**
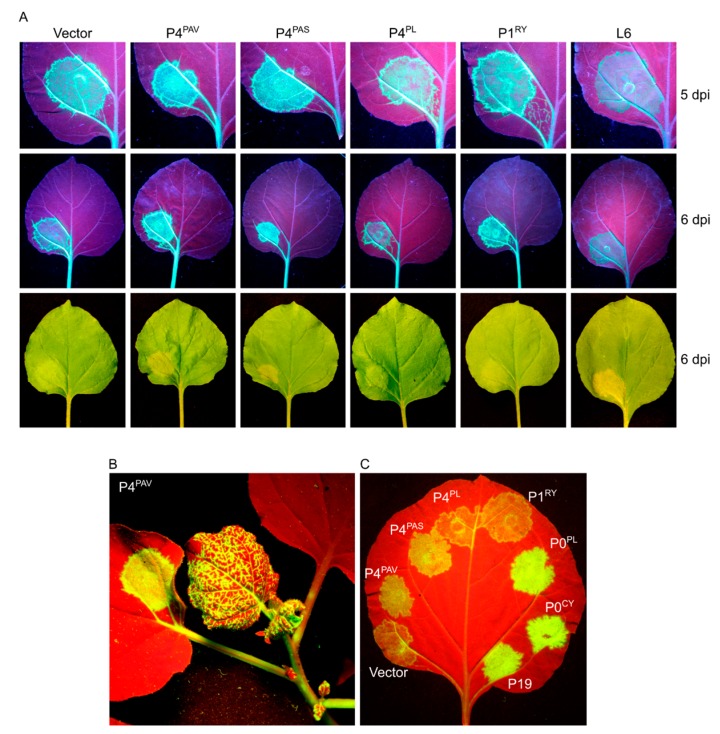
Effects of necrosis induced by luteovirus P4 on virus replication and spread. (**A**) Wild-type *N. benthamiana* plants were infiltrated with PVX tagged with GFP (PVX-GFP) plus empty vector (vector), BYDV-PAV P4 (P4^PAV^), BYDV-PAS P4 (P4^PAS^), PLRV P4 (P4^PL^), RYMV P1 (P1^RY^) or the fungal resistance gene L6. Pictures were taken either under UV or normal light at 5 and 6 days post-infection (dpi). (**B**) Systemic spread of PVX-GFP co-infiltrated with P4^PAV^ at 5 dpi. (**C**) Local silencing suppression assay by co-infiltration of PVX-GFP with the empty vector, P4^PAV^, P4^PAS^, P4^PL^, P1^RY^, PLRV P0 (P0^PL^), CYDV P0 (P0^CY^) or the TBSV P19.

To test whether the processes induced by P4^PAV^ and P4^PAS^ might allow viral replication and spread to occur before the death of infected cells, a PVX-GFP infectious clone was agroinfiltrated in various combinations with the P4 and control constructs into wild-type *N. benthamiana* leaves using the concentrations that induce the cell death response. As expected, agroinfiltration of the PVX-GFP construct with P4^PL^, P1^RY^, or vector backbone, which do not induce necrosis, produced PVX-GFP that, at 5 dpi, was able to replicate and spread from the infiltration spots to the nearest veins, then pass via the petiole (Figure 7A) to the apex of the plant (Figure 7B). Despite inducing tissue necrosis, the PVX-GFP/P4^PAV^ and PVX-GFP/P4^PAS^ infiltrations with agro-inoculum strength of 0.5 OD also generated PVX that was able to spread systemically at this same time point (Figure 7A,B). Moreover, even the co-infiltration of PVX-GFP with a fungal resistance gene (L6), which also induces cell death, allowed PVX to replicate and spread systemically at 6 dpi.

This assay with a GFP-encoding PVX construct also identifies local VSRs, since PVX-GFP replicates to a higher level and expresses its GFP gene more strongly and for longer in the presence of a VSR [20]. The co-infiltration of PVX-GFP with P4^PAV^ or P4^PAS^ constructs led to a slight increase in GFP fluorescence in infiltrated spots when compared to P4^PL^, P1^RY^, or vector backbone (Figure 7A,C), which confirms the weak local VSR activity by P4^PAV^ and P4^PAS^. Although the two luteovirus P4 proteins did not display strong local silencing suppression activity during PVX replication when compared to P0^PL^, P0^CY^ and P19 (Figure 7C), the replicated virus was still able to exit before the death of the infected cell, induced by P4.

## 4. Discussion

RNA silencing is the main anti-viral mechanism in plant tissues and the viral counter-defence usually involves avoidance or inactivation of the mechanism. As a survival strategy, the P0 proteins of some poleroviruses and the enamovirus have been reported to be strong local VSRs, acting by destabilizing AGO1 [20,44,45,49,50]. The viruses of the family *Luteoviridae* are strongly associated with the phloem tissues of their host plants [51], and the possession of strong locally-acting VSRs by the enamovirus and the poleroviruses might suggest that cells of the vasculature possess highly active RNA silencing machinery. The levels of virus-derived siRNAs detected by deep-sequencing PLRV- and cotton leafroll dwarf virus-infected materials (representing 15% and 6% of total sRNAs, respectively) support this [52,53]. However, the luteovirus BYDV-PAV replicates in cereals to similarly high levels as the polerovirus cereal yellow dwarf virus (CYDV)-RPV [33], and its siRNA profile revealed that it too is attacked by the RNA silencing machinery (this report). Yet, BYDV-PAV does not have an ORF in a counterpart genomic location to the enamovirus or poleroviruses, and testing all of the proteins expressed from the genome failed to identify any strong locally-acting VSR. 

A feature of BYDV-PAV that is not shared with poleroviruses is the production of a highly abundant subgenomic RNA3. It seemed possible that BYDV-PAV, in lieu of a strong locally-acting VSR, might express this non-coding RNA as a decoy for the RNA silencing machinery. CaMV has been shown to use such a strategy, producing an RNA from the leader sequence of its 35S transcript that attracts the plant’s siRNA generating machinery [54]. However, despite sgRNA3 accumulating to levels approaching 100-fold higher than the genomic RNA [33], there was not a dramatic overabundance of siRNAs produced from this genomic region. This shows that either sgRNA3 does not play this role or acts by augmenting the number of RNAs that AGO must scan within the cell, thus quenching its activity.

Remarkably, the protein previously identified as a movement protein, encoded by BYDV-PAV ORF 4, suppressed the spread of the silencing signal in *N. benthamiana* assays; a capacity also displayed by BYDV-PAS P4, but not polerovirus PLRV P4. The systemic VSR activity by the two luteovirus P4 proteins was accompanied by a reduced production of local siRNAs and a delay in the short-distance spread of the silencing signal, and these effects were dose-dependent. The systemic VSR activity by P0 proteins of the poleroviruses PLRV (P0^PL^), CYDV (P0^CY^) and sugarcane yellow leaf virus (P0^SC^) and the enamovirus PEMV-1 (P0^PE^) also induce similar effects, although they also have strong local VSR activity [20,49]. In the case of P0s, the destabilization of AGO1 provides a mechanism for the suppression of systemic spread of the silencing signal, since AGO1 appears to be required for systemic silencing [20,42,55,56]. Because luteoviruses lack an F-box-like P0 protein, it is possible that P4^PAV^ and P4^PAS^ operate differently to P0 to reduce the production or accumulation of siRNAs and suppress systemic silencing. The lack of strong local VSR activity by P4^PAV^ and P4^PAS^ compared to polerovirus and enamovirus P0s also supports this hypothesis. The degree of systemic silencing suppression by P4^PAV^ and P4^PAS^ was similar to RYMV P1, which is another example of a movement protein with effective systemic VSR activity and very weak local silencing suppression, but its suppression mechanism still remains largely unknown [57,58]. 

The expression of P4^PAV^ and P4^PAS^ in *N. benthamiana* elicits a response by the plant that leads to cell death in the infiltrated tissues, and the degree of necrosis directly correlates with P4 titers. Similar observations were recently reported for BYDV P4 [59]. Different poleroviral P0 proteins (P0^PL^, P0^CY^, P0^SC^ and beet western yellows virus P0) have also been reported to trigger necrosis in infiltrated tissues [20,49]. It could be hypothesised that P4^PAV^ and P4^PAS^ caused the infiltrated cells to die before the sGFP construct could be transcribed to high levels and before secondary siRNAs could be amplified from GFP mRNA, which would explain the low levels of GFP siRNAs in these tissues (Figure 6) and the reduction in red halo formation (Figure 3 and Figure 4). However, the inhibition of necrosis in infiltrated tissues, by concomitantly expressing the antiapoptotic protein CED-9 with P4^PAV^ (Figure 3 and Figure S2), did not affect the P4^PAV^ VSR activities of delaying the short-distance movement of the silencing signal (seen as reduced red halo) and displaying strong suppression of systemic silencing, nor did it significantly increase GFP fluorescence in the infiltrated spots. This suggests that the VSR activity displayed by P4^PAV^ and P4^PAS^ on the reduction of secondary siRNA production or accumulation is independent of the necrotic process. 

Luteoviruses are known to induce phloem necrosis in their hosts and it is possible that the P4 protein of these viruses could act as elicitors of this response. Nevertheless, the necrosis triggered by luteovirus P4 in infiltrated tissues did not inhibit P4’s systemic VSR activity against the propagation and spread of anti-viral siRNAs, nor did it prevent the replication and egress of PVX. Although lacking a strong local silencing suppressor, the strong systemic VSR activity by BYDV-PAV P4 and BYDV-PAS P4 and the weaker VSR activity by P6^PAV^, suggest that suppression of systemic silencing is the principal mechanism by which the luteoviruses BYDV-PAV and BYDV-PAS minimize the effects of the plant’s anti-viral defense.

## Supplementary Materials

The following are available online at www.mdpi.com/1999-4915/9/10/294/s1, Figure S1: Phylogenetic analysis of Luteovirus P6 proteins and luteovirid P4 proteins, Figure S2: Local systemic silencing assay with BYDV-PAV proteins P4 and P6.
