# Supplementary figures and images for: The Luteovirus P4 Movement Protein Is a Suppressor of Systemic RNA Silencing
Source: Viruses. 2017 Oct 10;9(10):294. doi: 10.3390/v9100294 (PMC5691645; doi:10.3390/v9100294)

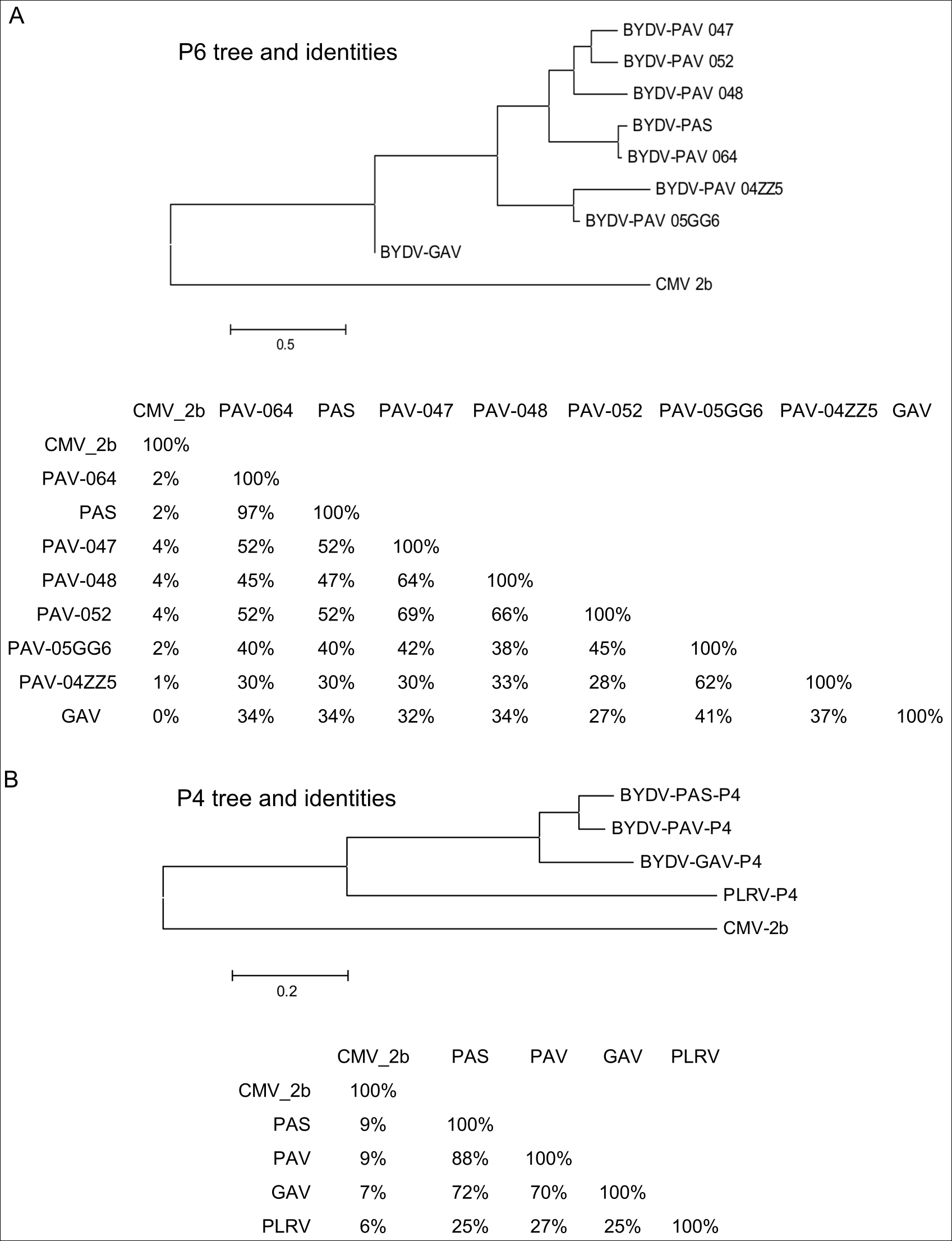

Supplement: Supplementary file 1 [file viruses-09-00294-s001.zip › FigS1_final.png]

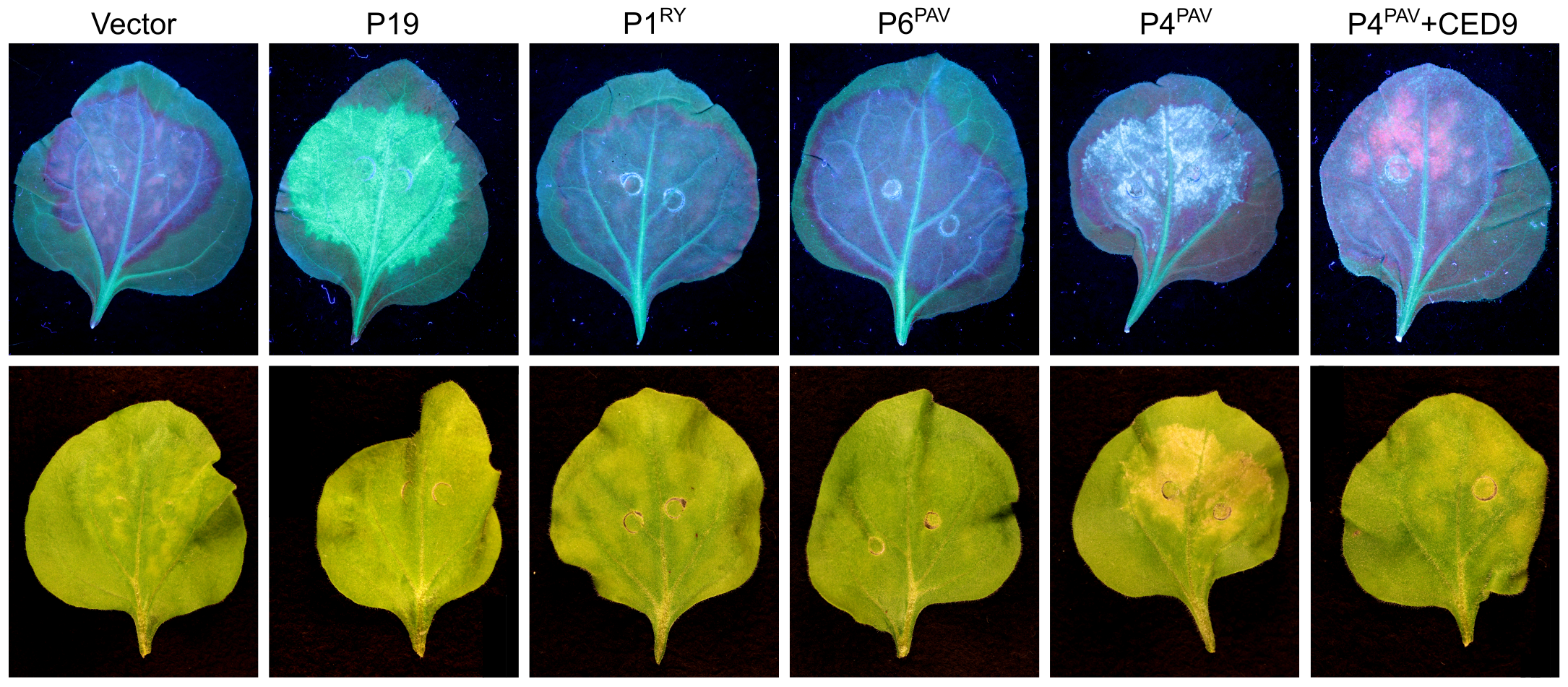

Supplement: Supplementary file 1 [file viruses-09-00294-s001.zip › FigS2_final.png]
